# Wills’ Hospital—Annual Report

**Published:** 1840-01-18

**Authors:** John Neill


### WILLS' HOSPITAL—ANNUAL REPORT.

BY JOHN NEILL.

This institution was founded by James Wills, a citizen of Philadelphia, for the relief of the indigent blind and lame. It commenced its operations in March, 1834, under the direction of a Board of eighteen Managers, six of whom were elected yearly by the City Councils, to serve for three years. The building is eligibly situated on Race street, between Schuylkill Fourth and Fifth streets, opposite Logan Square. The front is composed of sandstone, of a reddish hue, and is ornamented with six Ionic pilasters, supporting a proportionate entablature and pediment. The front door is in the principal story, and is approached by a flight of steps surmounted by a Grecian Ionic portico of four columns, the whole of which is composed of the same material as the front. The remaining part of the building is composed of rubblestone, and rough-cast, in imitation of the front. The whole interior of the house is divided into two parts—one for males, and the other for females. The basement story contains two dining-rooms, two bathing-rooms, two pantries, one large kitchen, a scullery, and a coal cellar.

In the principal story, on the right, is the lecture room, twenty-one by thirty-two feet,—on the left, the office, and steward's parlour, each twenty-one by sixteen feet,—and on the south side of the house, six dormitories, three on each side, and each ten by eleven feet. In the upper story, on the north, are two wards—one for females, thirty-two by twenty-one feet, and the other for males, forty-four by twenty-one feet; on the south side are seven dormitories, ten by eleven feet,—some are occupied by the household. The attic consists of two rooms together, extending the whole length of the building, which are at present useless—the house being illy constructed in a medical point of view. An important improvement was made in the summer by converting five rooms into the present wards, which secures a greater circulation of air, a more equable temperature, and a better discipline.

By the present arrangement, the hospital will contain forty-two beds for patients, and room for the requisite number of attendants. The whole building is roofed with copper, and heated by furnaces; each room has also a fire-place, and ventilator in the chimney. The stairways are composed of marble, and iron railings.

On the south side of the house there is a piazza enclosed with Venitian blinds, which protects the six rooms on the first floor from the glare of the sun, and also affords a pleasant place for exercise.

The officers consist of a steward and matron, together with the requisite number of nurses and servants, and a resident student or physician, who has the charge of the apothecary shop. The office of "Medical Resident" has been created within the last year. There are four surgeons and five physicians who attend in rotation, their period of service being three months.

A Visiting Committee of Managers sit on Wednesday and Saturday afternoons, to admit and discharge patients. No patient is admitted, unless it is thought by the surgeon or physician on duty that his case is susceptible of relief, so that no chronic or incurable cases are received into the house.

The number received during the last year is 80, and the number discharged 76. The following is a classified statistic of the discharged.

| | Cured. | Relieved. | Incurable. | Removed. |
|---|---|---|---|---|
| Eyes. | | | | |
| Amaurosis | 5 | 2 | 2 | 2 |
| Cataract | 2 | 0 | 0 | 0 |
| " false | 0 | 0 | 0 | 1 |
| Iritis | 1 | 0 | 0 | 0 |
| Obst. lachm. duct | 1 | 0 | 0 | 1 |
| Occlusion of pupil | 0 | 1 | 0 | 0 |
| Opacity of cornea | 1 | 1 | 0 | 0 |
| Ophthalmia | 3 | 0 | 0 | 0 |
| " acute | 9 | 1 | 0 | 0 |
| " catarrhal | 3 | 0 | 0 | 1 |
| " chronic | 2 | 0 | 0 | 1 |
| " granular | 7 | 1 | 0 | 3 |
| " purulent | 1 | 0 | 0 | 0 |
| " rheumatic | 1 | 1 | 0 | 1 |
| " scrofulous | 7 | 0 | 0 | 2 |
| " tarsal | 4 | 0 | 0 | 0 |
| Staphyloma | 1 | 0 | 0 | 0 |
| | 48 | 7 | 2 | 12 |
| Limbs. | | | | |
| Chorea | 1 | 0 | 0 | 0 |
| Diseased joints | 1 | 1 | 1 | 0 |
| Rheumatism | 2 | 1 | 0 | 0 |
| | 52 | 9 | 3 | 12 |
| Total, 76. | | | | |

It will be seen, by the above statement, that by far the greater number are afflicted with diseases of the eye, so that the institution is usually considered an ophthalmic hospital.

The number in the hospital at one time has averaged, during the past year, twenty-five. All were not on the poor list. Pay patients are received, who have comfortable accommodations, and are not subjected to the same regulations as those on charity.

A system of out-door relief was established a few months before the close of the year, which promises to be a source of much benefit to those who do not wish to enter a public institution. The number of applicants is as yet inconsiderable, it not being yet generally known that such a plan has been adopted, and that advice and medicines are furnished gratuitously.

The managers have also given the surgeons permission to introduce the system of clinical instruction. This will be advantageous to students and the profession, it being almost impossible to study diseases of the eye, except in a public institution. A course of clinical lectures is now being delivered in the lecture room of the building, and it is proposed to continue a course of clinical instruction during the year.

The students of this hospital will not only have the privilege of attending on prescribing days, but also of attending at the daily visit. Diseases of the eye cannot be studied by hastily following the steps of a professor through a general hospital: the student should see the cases frequently, and witness the daily applications, or he will not be able to recollect their condition and treatment.

*January 1st*, 1840.

